# Pathological mitochondria in neurons and perivascular astrocytic endfeet of idiopathic normal pressure hydrocephalus patients

**DOI:** 10.1186/s12987-019-0160-7

**Published:** 2019-12-18

**Authors:** Md Mahdi Hasan-Olive, Rune Enger, Hans-Arne Hansson, Erlend A. Nagelhus, Per Kristian Eide

**Affiliations:** 1 Department of Neurosurgery, Oslo University Hospital-Rikshospitalet, 0027 Oslo, Norway; 2 Institute of Clinical Medicine, Faculty of Medicine, University of Oslo, Oslo, Norway; 3 GliaLab and Letten Centre, Division of Physiology, Department of Molecular Medicine, Institute of Basic Medical Sciences, University of Oslo, 0317 Oslo, Norway; 4 Department of Neurology, Oslo University Hospital-Rikshospitalet, 0027 Oslo, Norway; 5 Institute of Biomedicine, University of Gothenburg, Göteborg, Sweden

**Keywords:** Mitochondria, Mitophagy, Endoplasmic reticulum, Autophagic vacuoles, Neurons, Astrocytes, Immunogold electron microscopy, Idiopathic normal pressure hydrocephalus

## Abstract

**Background:**

A growing body of evidence suggests that the accumulation of amyloid-β and tau (HPτ) in the brain of patients with the dementia subtype idiopathic normal pressure hydrocephalus (iNPH) is associated with delayed extravascular clearance of metabolic waste. Whether also clearance of intracellular debris is affected in these patients needs to be examined. Hypothetically, defective extra- and intra-cellular clearance of metabolites may be instrumental in the neurodegeneration and dementia characterizing iNPH. This study explores whether iNPH is associated with altered mitochondria phenotype in neurons and astrocytes.

**Methods:**

Cortical brain biopsies of 9 reference (REF) individuals and 30 iNPH patients were analyzed for subcellular distribution and morphology of mitochondria using transmission electron microscopy. In neuronal soma of REF and iNPH patients, we identified normal, pathological and clustered mitochondria, mitochondria-endoplasmic reticulum contact sites and autophagic vacuoles. We also differentiated normal and pathological mitochondria in pre- and post-synaptic nerve terminals, as well as in astrocytic endfoot processes towards vessels.

**Results:**

We found a high prevalence of pathological mitochondria in neuronal soma and pre- and post-synaptic terminals, as well as increased mitochondrial clustering, and altered number of mitochondria-endoplasmic reticulum contact sites in iNPH. Non-fused autophagic vacuoles were more abundant in neuronal soma of iNPH patients, suggestive of cellular clearance failure. Moreover, the length of postsynaptic densities was reduced in iNPH, potentially related to reduced synaptic activity. In astrocytic endfoot processes, we also found increased number, area and area fraction of pathological mitochondria in iNPH patients. The proportion of pathological mitochondria correlated significantly with increasing degree of astrogliosis and reduced perivascular expression of aquaporin-4 (AQP4), assessed by light microscopy immunohistochemistry.

**Conclusion:**

Our results provide evidence of mitochondrial pathology and signs of impaired cellular clearance in iNPH patients. The results indicate that iNPH is a neurodegenerative disease with close similarity to Alzheimer’s disease.

## Background

Idiopathic normal pressure hydrocephalus (iNPH) is a neurodegenerative disease and subtype of dementia, clinically characterized by gait ataxia, urinary incontinence, and cognitive decline [1]. A special feature of this condition is that symptoms may be improved by cerebrospinal fluid (CSF) diversion surgery, at least to some extent and for a period of time [2, 3], due to the CSF circulation disturbance characterizing the condition. The cause of iNPH remains unknown, but there is an increasing awareness of the overlap between iNPH and Alzheimer’s diseases [4]. A significant proportion of iNPH patients presents with extra-cellular accumulation of amyloid-β, and intracellular accumulation of hyperphosphorylated tau (HPτ) [5–7]. Both are hallmarks of Alzheimer’s disease, related to defective clearance of the brain metabolites.

Since 2012 the glymphatic system has emerged as an important pathway for clearance of brain metabolites [8, 9], though it is still debated [10]. The glymphatic system involves CSF influx along cerebral arteries, convective flow of fluid and waste through the brain parenchyma, and drainage along veins [9, 11]. An essential feature of glymphatic clearance is its dependency on the water channel aquaporin-4 (AQP4) [8], which is enriched in astrocytic endfoot processes surrounding cerebral vessels [12]. Evidence suggests that glymphatic function may become impaired with increasing age [13], and in Alzheimer’s disease [9]. With regard to iNPH, recent imaging studies have provided evidence for delayed glymphatic clearance of molecules from the extravascular space of the entire brain [14–16].

While defective glymphatic clearance may underlie the extracellular accumulation of amyloid-β in iNPH, it has not been demonstrated whether cellular clearance processes are impaired as well.

The present study was undertaken to explore whether iNPH patients provide evidence of altered mitochondrial phenotype, denoted pathological mitochondria, which may be indicative of impaired cellular metabolism and clearance processes in iNPH. We focused on neuronal soma, pre- and postsynaptic terminals and perivascular astrocytic endfeet processes, and examined various measures of mitochondria, and autophagy. We provide evidence of clear mitochondrial pathology in iNPH. The observations of pathological mitochondria correlated with other features of iNPH like astrogliosis, loss of perivascular AQP4, and even altered pulsatile intracranial pressure (ICP).

## Materials and methods

### Ethical approvals

The study was done according to the following approvals: Regional Committee for Medical and Health Research Ethics (REK) of Health Region South-East, Norway: (i) Brain Tissue Research Biobank (Approval no. REK 2010/1030 and 2012/1157). (ii) Brain biopsy from iNPH patients (Approval no. REK 2009/2060). (iii) Storage of brain tissue from REF patients, i.e. patients undergoing neurosurgery for various reasons (epilepsy, tumor or cerebral aneurysms) wherein removal of brain tissue is necessary as part of treatment (Approval no. REK 2011/2306). Oslo University Hospital (Approvals no. 10/6806 and 2011/19311). The study was performed in accordance with the ethical standards as laid down in the 1964 Declaration of Helsinki and its later amendments or comparable ethical standards. Consecutive patients were included after oral and written informed consent.

### Experimental design

The study design was prospective and observational: Randomization of patients was not relevant; neither was a priori sample size calculation needed. The person analyzing the electron microscopy specimens (MMHO) was blinded to patient data and diagnosis.

### Participants

#### REF individuals

Apparently normal brain tissue was sampled from patients undergoing neurosurgery for various reasons, and in whom removal of normal brain tissue was required as part of the planned brain surgery (REF individuals): (i) Patients undergoing brain tissue resection for epilepsy (6 patients). (ii) Patients undergoing elective clipping of cerebral aneurysm (2 patients). (iii) Patients undergoing tissue resection for brain tumor (1 patient).

#### iNPH patients

Consecutive iNPH patients (n = 30) referred to the Department of neurosurgery, Oslo University Hospital-Rikshospitalet, Oslo, Norway, were included based on symptoms and clinical findings indicative of iNPH and imaging findings of ventriculomegaly. The diagnostic work-up of iNPH patients included a 3-day stay in the neurosurgery department. Day 1 (day of admittance), clinical neurological assessment and magnetic resonance imaging (MRI) were performed if not done recently. Day 2, an intracranial pressure (ICP) sensor was implanted and brain biopsy performed. Day 3, following over-night ICP monitoring, it was determined whether the patient should be offered surgical treatment. Clinical severity of iNPH was graded according to a previously described NPH grading scale (15 possible scores ranging from 3 to 15), which assesses the combined severity of gait disturbance, urinary incontinence and dementia [2, 3].

The indication for shunt surgery was based on the combination of clinical findings, presence of co-morbidity, imaging findings and results of the ICP monitoring. Clinical improvement following surgery was assessed in the out-patient clinic, and severity graded using the NPH grading scale [2, 3].

### Sampling and handling of brain tissue

The sampling of brain tissue was done in conjunction with placement of an ICP sensor for overnight ICP monitoring. The diagnostic procedure was done in the operation room. In local anesthesia, a skin incision was done frontally on the right side, a small burr hole about 1 cm in diameter was made, the dura opened, and the brain surface exposed. A disposable Nashold Biopsy Needle (Integra Radionics, Burlington, MA, USA) was introduced immediately below the cortical surface and a biopsy (0.9 mm × 10 mm) aspirated through the needle. Then, a solid ICP sensor (Codman MicroSensor™, Johnson & Johnson, Raynham, MA, USA) was tunneled subcutaneously, zeroed against the atmospheric pressure and introduced 1–2 cm into the frontal cortex parenchyma via the small opening that was established by the biopsy. The overnight monitoring and subsequent analysis of ICP scores was undertaken as described in detail before [2, 3].

### Post embedding electron microscopy

The detailed technical procedures for the handling, processing and analysis of the frontal cortex biopsies have been reported [17, 18]. In brief, the dissected brain tissue was fixed in the operating room to avoid time delay by immersion in 0.1 M phosphate buffer containing 4% paraformaldehyde and 0.25% glutaraldehyde and cut into small specimens (typically 0.5 mm × 0.5 mm × 1 mm). The electron microscopy specimens were subjected to freeze substitution and infiltration in Lowicryl HM20 resin (Polysciences Inc., Warrington, PA, USA, Cat 15924). A Reichert ultramicrotome (Reichert Techn., Wien, Austria) was used for cutting sections of 80 nm thicknesses, which were mounted on nickel grids and further processed for counterstaining with 1% uranyl acetate and 0.3% lead citrate for ultrastructure analysis.

### Mitochondrial quantitative assessment

The electron micrographs were recorded with a FEI Tecnai™ 12 transmission electron microscope (FEI Company, Hillsboro, OR, USA) at different magnifications of 43,000×, 26,500×, 20,500×, 11,500×, 9900×, 8200×, 6000×, and 4200×. Electron micrographs were saved as 8-bit, 2048 × 2048 pixels tagged image format images (TIFFS). Images were subsequently analyzed using a customized version of a MATLAB toolbox [19] designed for evaluation of mitochondrial area (Additional file 1: Fig. S1). Images were segmented manually by drawing polygonal selections over identifiable cellular compartments and structures. Neuronal soma, and pre- and post-synaptic terminals, and astrocytic endfoot processes were identified and outlined.

The number of mitochondria per patient; area of mitochondria per neuronal soma in each patient, and mitochondrial area fraction (area covered by mitochondria divided by total cytoplasmic area).

### Mitochondrial qualitative assessment

We classified all mitochondria systematically into three categories based on the following qualitative features:

- Normal mitochondria. These were dark and electron dense, with intact matrix and cristae, and regular shape.
- Pathological mitochondria. These were light and less electron dense, with less intact matrix cristae, and irregular shape and a swollen appearance.
- Clustered mitochondria. These were clumped and aggregated due to accumulation of damaged mitochondria. Mitochondria were irregularly electron dense, with less intact cristae, and of irregular shape. The presence of clustered mitochondria is assumed to reflect defective “mitophagy” [20].

A cartoon illustrating the differentiation of normal, pathological and clustered mitochondria is shown in Additional file 1: Fig. S2.

### Other analyses

The number, area and fraction of autophagic vacuoles were examined in a similar fashion as the mitochondrial analysis (Additional file 1: Fig. S1). The length of postsynaptic densities of asymmetric synapses and length of mitochondria-endoplasmic reticulum contact sites were measured interactively in the MATLAB program (Additional file 1: Fig. S1).

### Light microscopy

We performed light microscopic immunohistochemistry and semi-quantitative assessment of glial fibrillary acidic protein (GFAP), aquaporin-4 (AQP-4), dystrophin 71 (Dp71), and Cluster of Differentiation 68 (CD68), as previously described [17, 21].

### Statistical analysis

Statistical analyses were performed using the SPSS software version 25 (IBM Corporation, Armonk, NY, USA). For each individual, the data were pooled and presented as mean ± standard deviation. Differences between categorical data were determined using Pearson Chi-square test, and differences between continuous data were determined using independent samples t-tests, Mann–Whitney U-test, or multivariate analysis to take into consideration age differences between groups. Correlation between variables was determined using Pearson correlation coefficient. Statistical significance was accepted at the 0.05 level (two-tailed).

### Data availability statement

The data presented in this work is available upon request.

## Results

### Patients

The patient material consists of 9 reference (REF) individuals and 30 iNPH patients. Demographic information is presented in Additional file 1: Table S1. The patient cohorts were different regarding age, co-morbidity (presence of diabetes), disease duration and severity of iNPH symptoms.

Biopsies from the 30 iNPH patients were from grey matter of frontal cortex, while REF biospies were from the grey matter of temporal cortex in four epilepsy cases, frontal cortex in three patients (2 aneurysm, 1 tumor), parieto-occipital cortex in one epilepsy case, and the occipital cortex in another epilepsy patient.

### Normal and pathological mitochondria in neurons

#### Neuronal soma

**Fig. 1:**
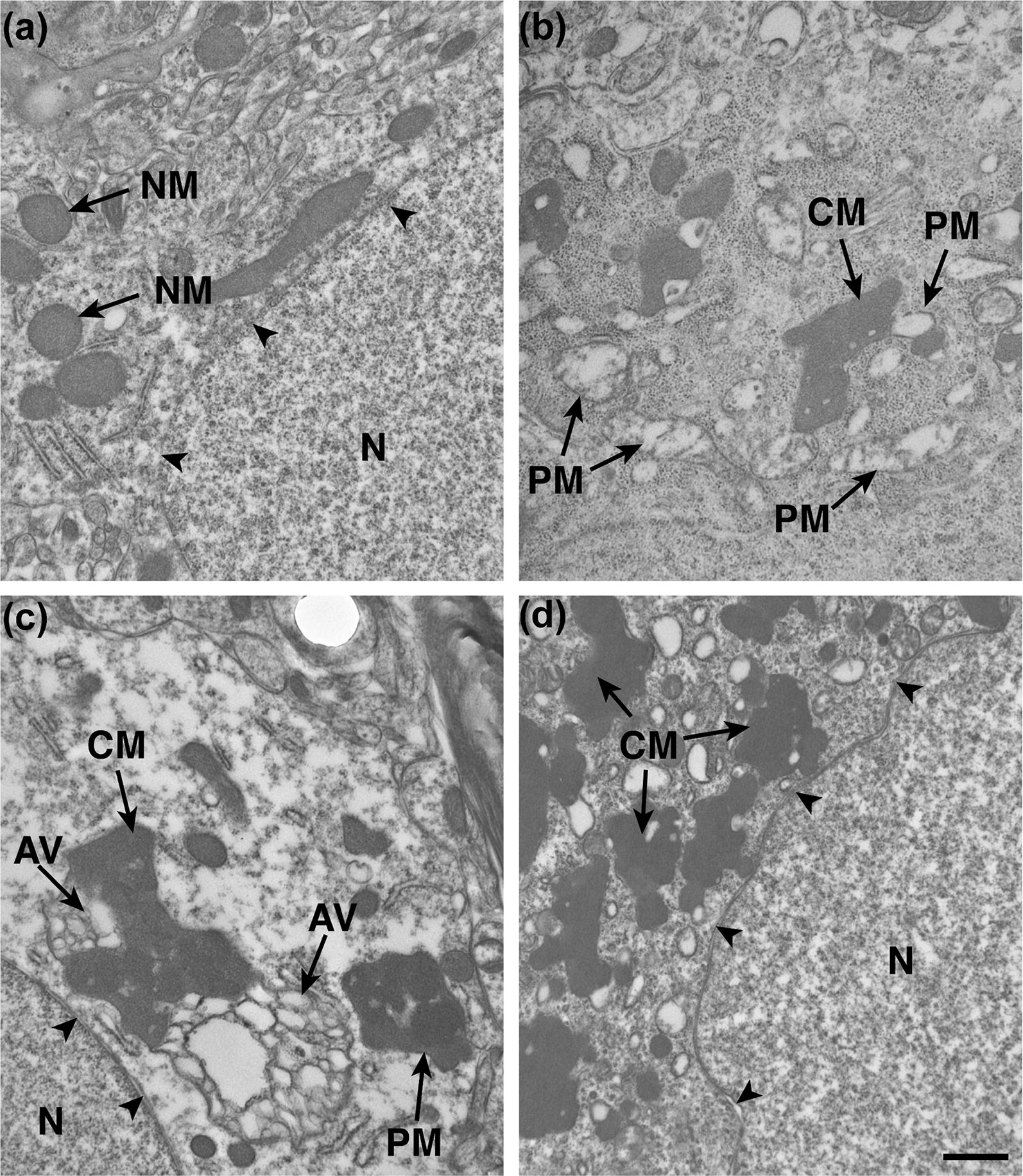
Normal, pathological and clustered mitochondria in neuronal soma. **a** Normal mitochondria (NM) with electron dense mitochondria in a REF individual. Nuclear membrane of nucleus (N) indicated by black arrowheads. **b** Clustered mitochondria (CM) with less electron dense cristae in soma of iNPH. **c** Pathological mitochondria (PM) engulfed by non-fused autophagic vacuoles (AVs). **d** Clustered mitochondria (CM) in the soma of iNPH. Magnification ×11,500; scale bar: 1 µm

From each individual of the REF and iNPH cohorts, we examined on average 12.7 ± 6.5 and 12.1 ± 5.8 soma, which included a mean cytoplasmic area of 16,982,978 ± 5,335,206 nm^2^ and 21,797,964 ± 7,383,110 nm^2^, respectively (not significantly different). Mitochondrial characteristics in soma of neurons are given in Table 1. While the fraction of normal mitochondria was significantly reduced in iNPH, the number of pathological mitochondria was significantly increased (Table 1). Examples of normal, pathological and clustered mitochondria in neuronal soma are given in Fig. 1. Further examples of normal mitochondria in REF subjects are shown in Additional file 1: Fig. S3, and of normal and pathological mitochondria in iNPH patients in Additional file 1: Fig. S4. Examples of clustered mitochondria in iNPH are given in Additional file 1: Fig. S5. The presence of clustered mitochondria, an indicator of mitochondrial abnormality, was significantly increased in iNPH (Table 1).

**Table 1:** Normal, pathological and clustered mitochondria in neuronal soma of REF and iNPH subjects

	REF	iNPH	Significance
Normal mitochondria			
Number of normal mitochondria per soma (N)	50.8 ± 31.2	36.8 ± 27.6	ns
Area of normal mitochondria per soma (nm^2^)	134,348 ± 28,130	122,202 ± 71,967	ns
Fraction of normal mitochondria per soma (%)	0.9 ± 0.3	0.6 ± 0.3	P = 0.014
Pathological mitochondria			
Number of pathological mitochondria per soma (N)	8.8 ± 11.2	50.9 ± 37.5	P = 0.004
Area of pathological mitochondria per soma (nm^2^)	240,857 ± 85,877	191,807 ± 77,411	ns
Fraction of pathological mitochondria per soma (%)	0.86 ± 0.73	0.94 ± 0.49	ns
Clustered mitochondria			
Number of clustered mitochondria per soma (N)	0.6 ± 1.3	13.6 ± 12.0	P = 0.007
Area of clustered mitochondria per soma (nm^2^)	535,546 ± 238.686	977,784 ± 501,167	P = 0.005
Fraction of clustered mitochondria per soma (%)	0.7 ± 1.5	4.7 ± 3.3	P = 0.04

Data presented as mean ± standard deviation (std). Significant differences between continuous variables were determined by multivariate analysis

#### Presynaptic terminals

From each individual of the REF and iNPH cohorts, we examined on average mean 38.9 ± 24.6 and 42.1 ± 18.1 presynaptic terminals (non-significant differences). The average area per individual of examined pre-synaptic terminals were 799,242 ± 216,666 nm^2^ and 781,147 ± 163,386 nm^2^, respectively (non-significant difference). In iNPH, the number of pathological mitochondria was significantly increased (Table 2).

**Table 2:** Normal and pathological mitochondria in pre- and postsynaptic terminals of REF and iNPH subjects

	REF	iNPH	Significance
Presynaptic terminals			
Normal mitochondria			
Number of normal mitochondria per presynaptic terminal (N)	25.6 ± 16.1	11.3 ± 5.3	ns
Area of normal mitochondria per presynaptic terminal (nm^2^)	91,017 ± 32,840	108,668 ± 81,513	ns
Fraction of normal mitochondria per presynaptic terminal (%)	11.9 ± 4.7	13.5 ± 9.6	ns
Pathological mitochondria			
Number of pathological mitochondria per presynaptic terminal (N)	0.1 ± 0.3	4.9 ± 4.2	P = 0.05
Area of pathological mitochondria per presynaptic terminal (nm^2^)	59,537 ± 178,611	152,763 ± 149,111	ns
Fraction of pathological mitochondria per presynaptic terminal (%)	7.2 ± 21.5	15.7 ± 6.4	ns
Postsynaptic terminals			
Normal mitochondria			
Number of normal mitochondria per postsynaptic terminal (N)	6.1 ± 5.6	2.9 ± 2.7	ns
Area of normal mitochondria per postsynaptic terminal (nm^2^)	160,597 ± 83,189	101,246 ± 83,823	P = 0.02
Fraction of normal mitochondria per postsynaptic terminal (%)	26.8 ± 15.1	15.2 ± 12.4	ns
Pathological mitochondria			
Number of pathological mitochondria per postsynaptic terminal (N)	0	3.1 ± 2.7	P = 0.02
Area of pathological mitochondria per postsynaptic terminal (nm^2^)	0	158,834 ± 109,688	P = 0.03
Fraction of pathological mitochondria per postsynaptic terminal (%)	0	33.0 ± 20.4	P = 0.02

Data presented as mean ± standard deviation (std). Significant differences between continuous variables were determined by multivariate analysis

#### Postsynaptic terminals

From each subject of the REF and iNPH cohorts, we examined on average 35.9 ± 23.2 and 40.4 ± 18.5 postsynaptic terminals (non-significant difference), which included an average area of 628,564 ± 208,871 nm^2^ and 515,394 ± 116,306 nm^2^ post-synaptic terminals, respectively (non-significant difference). In iNPH patients, the area of normal mitochondria per postsynaptic terminal was significantly reduced, while the number, area and fraction of pathological mitochondria per postsynaptic terminal were significantly increased (Table 2).

#### Ratio between mitochondria in soma versus pre- and postsynaptic terminals

**Fig. 2:**
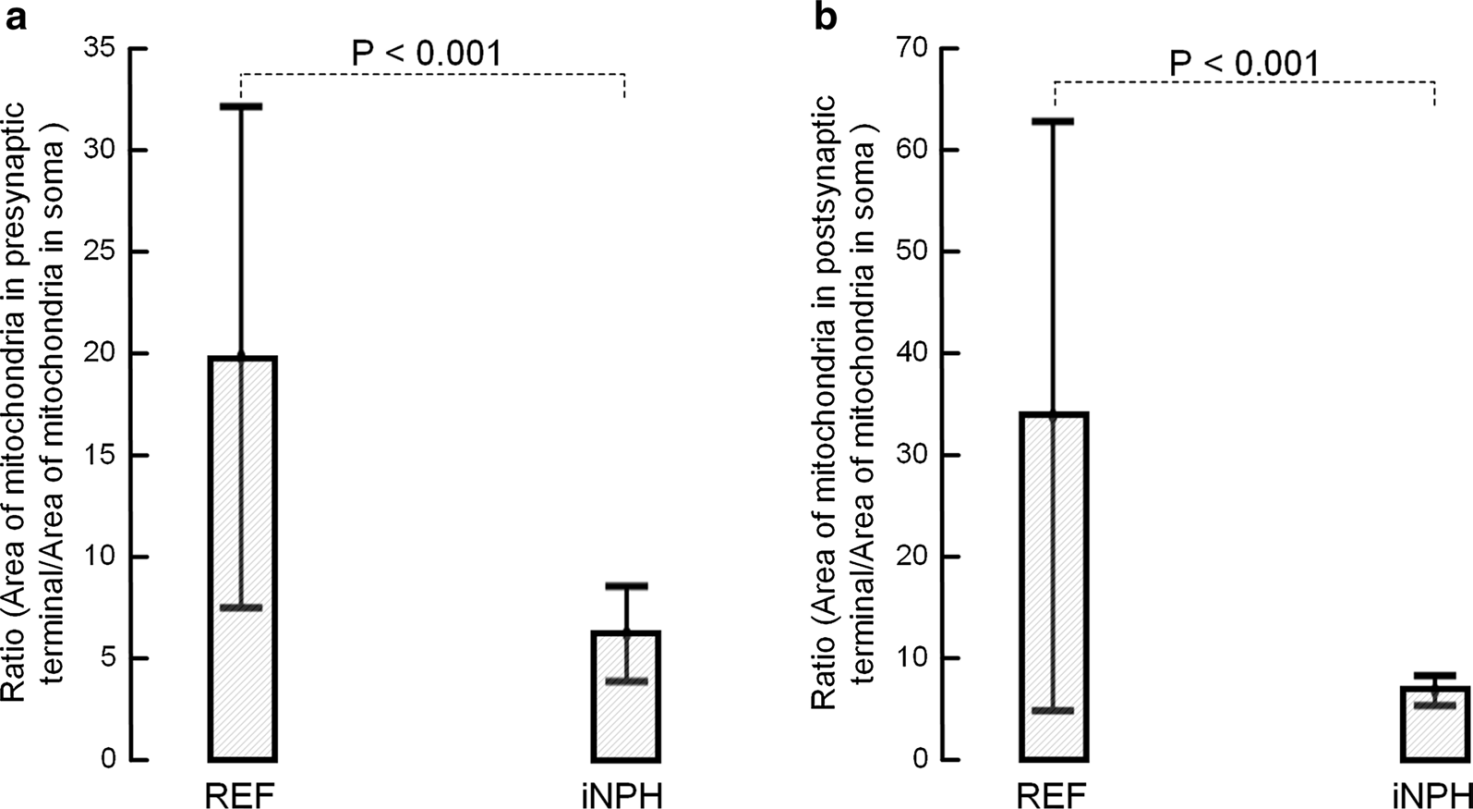
The ratio between areas of mitochondria in presynaptic terminals versus soma and the ratio between areas of mitochondria in postsynaptic terminals versus soma are lower in iNPH. **a** The ratio between area of mitochondria in presynaptic terminals and area of mitochondria in soma was significantly reduced in iNPH vs. REF subjects (16.8 ± 13.7 vs. 5.2 ± 3.6 in REF and iNPH, respectively. P < 0.001; multivariate analysis). **b** The ratio between area of mitochondria in postsynaptic terminals and area of mitochondria in soma was significantly reduced in iNPH vs REF subjects (33.6 ± 37.9 vs. 6.4 ± 2.7 in REF and iNPH, respectively. P < 0.001; multivariate analysis). Error bars are 95% CI

The ratio between mitochondria area in soma versus pre- and postsynaptic terminals is shown in Fig. 2. In iNPH, the ratio was significantly lower both for the pre- (Fig. 2a) and postsynaptic (Fig. 2b) terminals.

### Altered mitochondria-endoplasmic reticulum contacts in iNPH

**Fig. 3:**
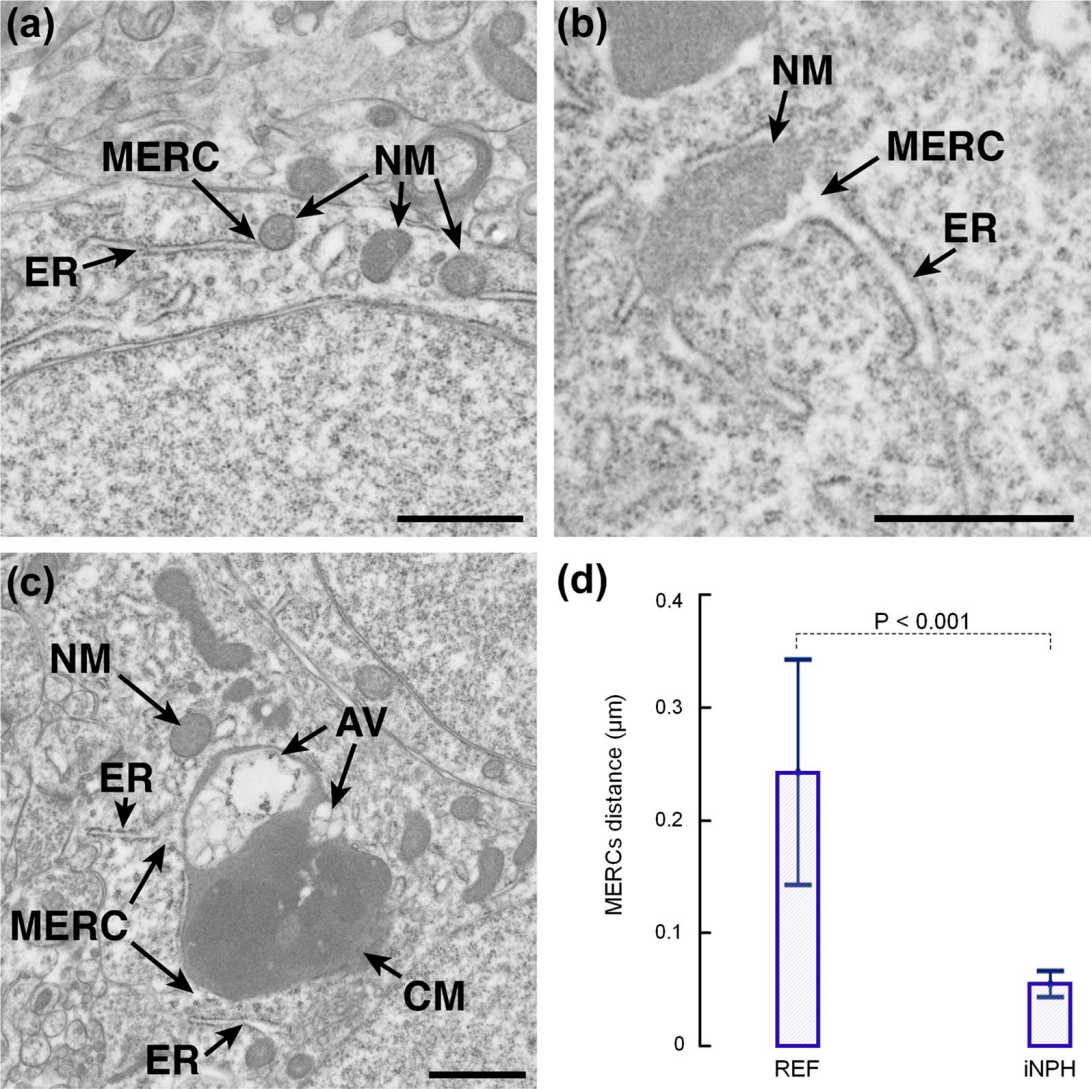
The distance between mitochondria and endoplasmic reticulum is reduced resulting in increased number of MERCs in iNPH. **a** The endoplasmic reticulum (ER) and normal mitochondria (NM) are shown in a REF individual, also demonstrating the MERCs. **b** In an iNPH patient the MERCs is indicated. **c** The MERCs distance was shorter in iNPH. **d** At group level, the MERCs was 4.4-fold longer in REF than iNPH individuals (0.24 ± 0.04 µm vs. 0.06 ± 0.03 µm; P < 0.001; multivariate analysis). The length of ER was similar in the REF versus iNPH cohorts (0.67 ± 0.30 µm vs. 0.78 ± 0.43 µm, P = 0.65). Error bars are 95% CI. Magnification ×11,500; Scale bar 1 µm. *CM* clustered mitochondria

The distance between endoplasmic reticulum (ER) and mitochondria was used as a measure of mitochondria-endoplasmic reticulum contact sites, and this distance was significantly reduced in iNPH patients, as illustrated in Fig. 3. The lengths of ER examined in these cohorts were comparable (0.67 ± 0.30 nm^2^ and 0.79 ± 0.43 nm^2^ in REF and iNPH patients, non-significant difference).

### Autophagy

**Fig. 4:**
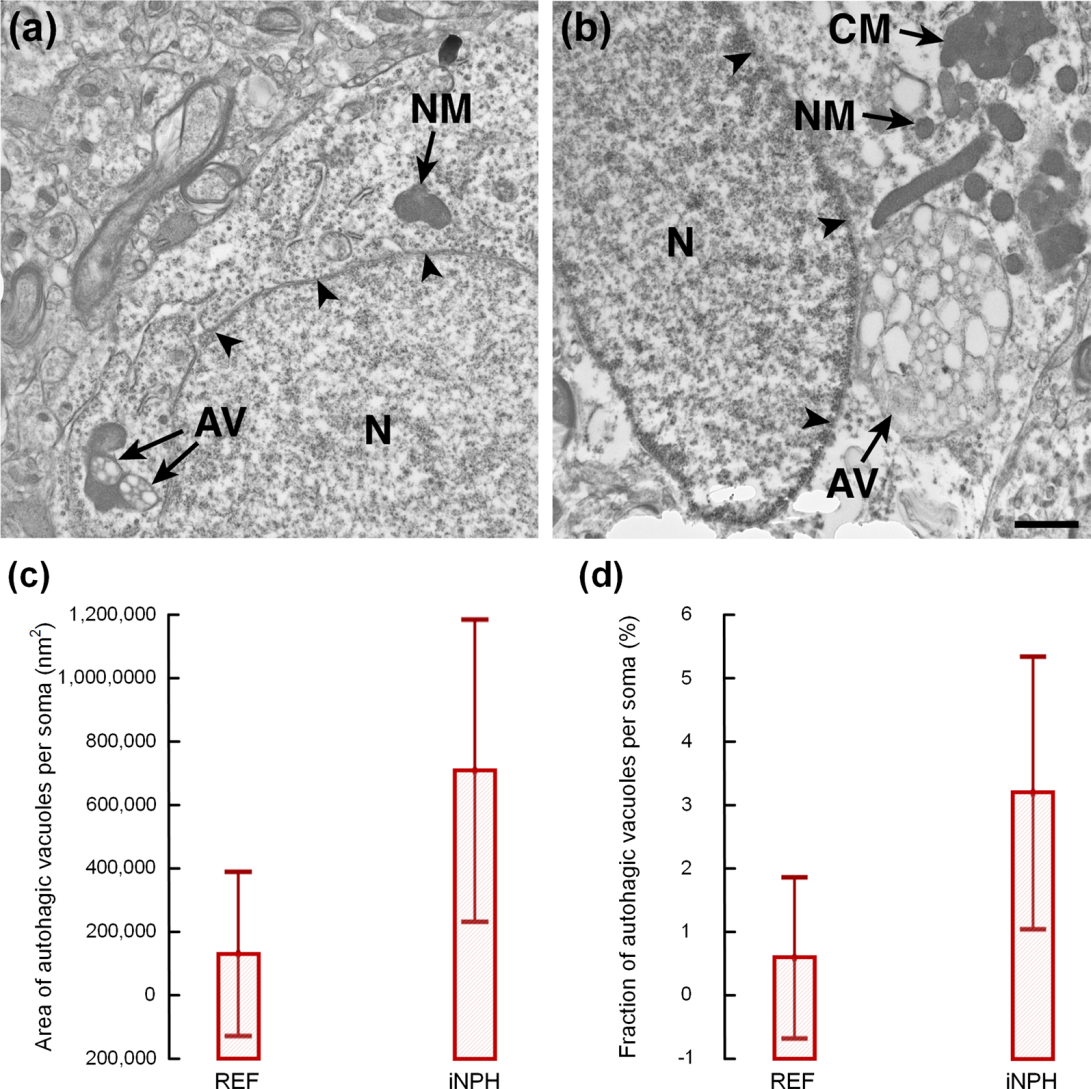
Tendency towards increased occurrence of autophagic vacuoles in iNPH. **a** Examples of autophagic vacuoles (AV) are given from **a** REF and **b** iNPH individuals. While autophagic vacuoles were observed in 2/9 (22%) REF individuals, it was seen in 16/28 (57%) iNPH patients (P = 0.068, Chi square test). **c** The area of autophagic vacuoles per soma was 5–6-fold higher in iNPH (125,393 ± 342,944 versus 713,706 ± 1,276,033 nm^2^; P = 0.069; Mann–Whitney U-test). Differences between groups were not significant when considering age-differences by multi-variate analysis. **d** The fraction of autophagic vacuoles was about fivefold higher in iNPH (0.6 ± 1.7 versus 3.2 ± 5.6; P = 0.04; Mann–Whitney U-test). However, multi-variate analysis showed no significant differences between groups when considering the age-differences. Error bars in **b** and **c** are 95% CI. Magnification ×11,500; Scale bar 1 µm. *N* nucleus. The nuclear membrane in indicated by black arrowheads

With regard to autophagy, we examined in individuals of REF and iNPH cohorts on average 12.7 ± 6.5 and 12.1 ± 5.8 soma, which included mean cytoplasmic areas of 16,982,978 ± 5,335,206 nm^2^ and 21,797,964 ± 7,383,110 nm^2^, respectively. Autophagy vacuoles (Fig. 4a, b) were seen in 2/9 REF individuals and in 16/28 iNPH cases (P = 0.068, Chi square test). The two REF individuals presenting autophagy vacuoles had long-lasting epilepsy (7 and 30 years durations), and both reported some cognitive impairment. Compared with REF subjects, the area of autophagy vacuoles per soma was 5–6-fold higher in iNPH (Fig. 4c), and the area fraction of autophagic vacuoles per soma was about fivefold higher in iNPH (Fig. 4d). Differences between groups were, however, not significant when taking into account the age differences between groups. Further examples of autophagic vacuoles are given in Additional file 1: Fig. S6.

### Post-synaptic density

**Fig. 5:**
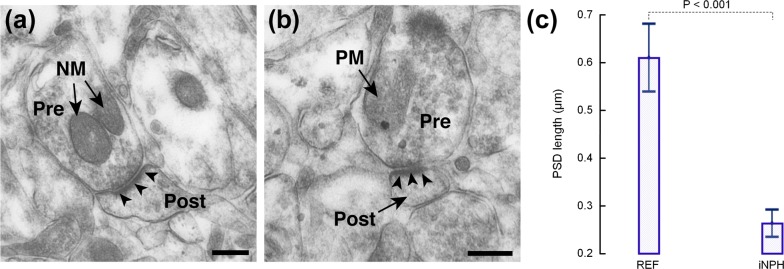
Reduced post-synaptic length in iNPH. Post-synaptic density (PSD) length (indicated by white arrow heads) is reduced in iNPH. **a** Electron microscopy from a REF individual showing presynaptic (Pre) and postsynaptic (Post) terminals, and PSD (black arrowheads). **b** The PSD in a postsynaptic (Post) terminal of an iNPH patient is indicated by black arrowheads. The PSD was 2.4-fold higher in REF than iNPH individuals (0.61 ± 0.09 µm vs 0.26 ± 0.06 µm (P < 0.001; multivariate analysis). Magnification ×11,500; Scale bar 1 µm. Error bars are 95% CI. *NM* normal mitochondria, *SM* pathological mitochondria

The post-synaptic density differed between iNPH and REF individuals (Fig. 5). Hence, the post-synaptic density length was on average 0.26 ± 0.06 µm in iNPH as compared to 0.61 ± 0.09 µm in REF subjects (P < 0.001; Fig. 5c).

### Normal and pathological mitochondria in astrocytic endfoot processes

In biopsies from individuals of the REF and iNPH cohorts, we examined mean 18.0 ± 8.2 and 20.3 ± 10.4 astrocytic endfoot processes, respectively (not significantly different). The average area of endfoot processes were 2,040,461 ± 1,009,156 nm^2^ and 1,587,893 ± 641,170 nm^2^, respectively.

**Fig. 6:**
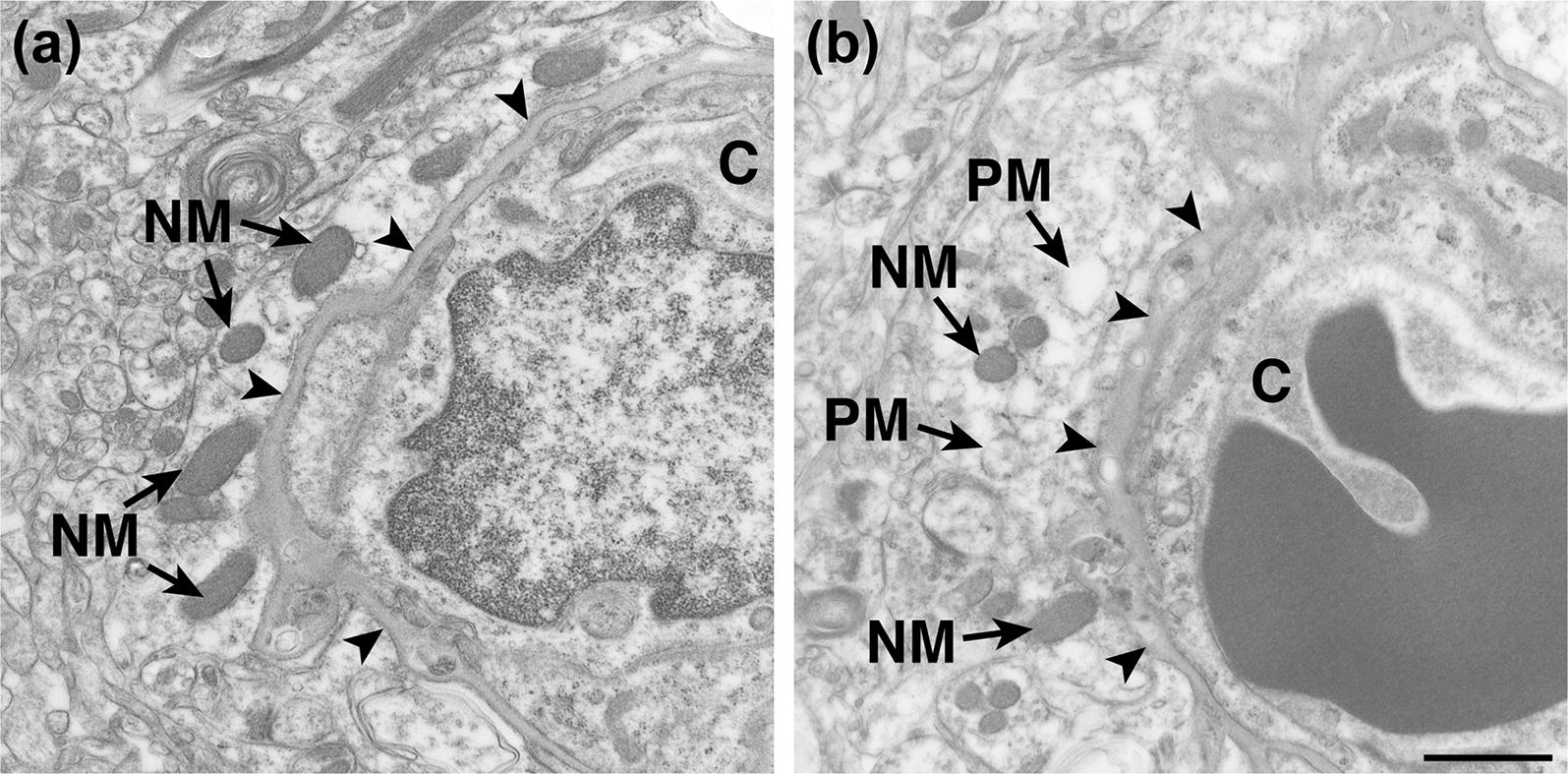
Increased proportion of pathological mitochondria in astrocytic endfeet of iNPH patients. Electron micrograph showing **a** the presence of normal mitochondria (NM) in the astrocytic endfeet in REF subjects, while **b** the number of pathological mitochondria (PM) was increased in iNPH patients. Basement membrane indicated by black arrowheads. Magnification ×16,500; scale bar 1 µm. *C* capillary lumen

Figure 6 shows normal mitochondria in astrocytic endfeet of REF subjects (Fig. 6a) and normal and pathological mitochondria in astrocytic endfeet of iNPH patients (Fig. 6b). The number of normal mitochondria in astrocytic endfoot processes was significantly reduced in iNPH (Table 3). Increased mass of pathological mitochondria in astrocytic endfoot processes of iNPH individuals was revealed by significantly increased number and area of pathological mitochondria (Table 3).

**Table 3:** Normal and pathological mitochondria in astrocytic endfoot processes of REF and iNPH subjects

	REF	iNPH	Significance
Normal mitochondria			
Number of normal mitochondria per astrocytic endfoot process (N)	18.4 ± 9.4	2.6 ± 3.6	P < 0.001
Area of normal mitochondria per endfoot processes (nm^2^)	132,672 ± 48,823	81,923 ± 82,860	ns
Fraction of normal mitochondria per endfoot process (%)	7.2 ± 3.0	5.3 ± 5.6	ns
Pathological mitochondria			
Number of pathological mitochondria per astrocytic endfoot process (N)	0.4 ± 0.8	3.8 ± 2.5	P = 0.003
Area of pathological mitochondria per endfoot processes (nm^2^)	28,903 ± 49,387	146,356 ± 86,682	P = 0.03
Fraction of pathological mitochondria per endfoot process (%)	1.8 ± 3.1	10.8 ± 8.4	ns

Data presented as mean ± standard deviation (std). Significant differences between continuous variables were determined by multivariate analysis

**Fig. 7:**
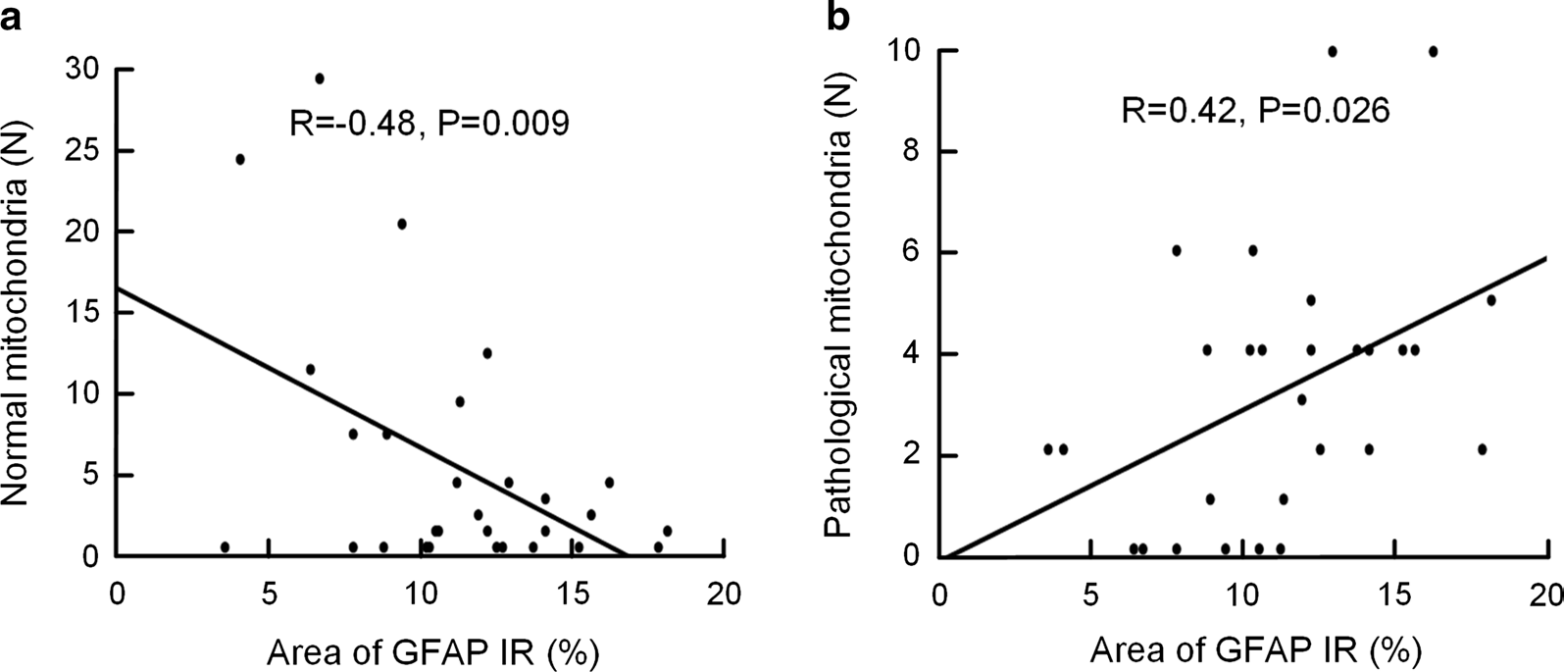
Significant association between degree of astrogliosis and number of normal and pathological mitochondria in astrocytic endfoot processes of REF and iNPH patients. **a** There was a significant negative Pearson correlation between percentage GFAP IR, measured by immunohistochemistry, and number of normal mitochondria in astrocytic endfoot processes (R = − 0.48, P = 0.009). **b** There was a positive correlation between degree of astrogliosis and number of pathological mitochondria in astrocytic endfoot processes (R = 0.42; P = 0.026)

With increasing astrogliosis, measured as high GFAP immunohistochemical labelling on light microscopic examinations, there was significantly reduced number of normal mitochondria (Fig. 7a) and significantly increased number of pathological mitochondria (Fig. 7b).

By using immunofluorescence, we also examined how the number of normal and pathological mitochondria related to the expression of other key molecules of the perivascular endfeet (Table 4). For all individuals of the iNPH and REF cohorts, the number of normal mitochondria was significantly and positively correlated with the expression of perivascular AQP4 and Dp71, while expression of AQP4 and Dp71 in neuropil was not correlated with number of normal mitochondria in the endfoot processes. On the other hand, the number of pathological mitochondria was significantly and negatively correlated with expression of AQP4 perivascularly and in neuropil, and significantly and negatively correlated with Dp71 perivascular (Table 4). Reduced perivascular expression of AQP4 was also significantly correlated with increased area of autophagic vacuoles in neuronal soma (Additional file 1: Fig. S7). It should be noted that the AQP4 expression referred to in Table 4 and Additional file 1: Fig. S7 refers to arbitrary units (AU); reduced arbitrary units (AU) is indicative of increased AQP4 expression because higher AQP4 causes less transmission of light and thereby reduction of arbitrary units. With regard to expression of CD68, a marker of inflammation, higher number of normal mitochondria seemed to be associated with lower expression of CD68 (P = 0.056; Table 4).

**Table 4:** Association between number of normal and pathological mitochondria in astrocytic endfoot processes and light microscopy findings of the REF (n = 9) and iNPH (N = 30) cohorts

Light microscopy immunohistochemistry	Mitochondria in astrocytic endfoot processes	
	Number of normal mitochondria	Number of pathological mitochondria
AQP4-perivascular (AU)	R = − 0.61, P < 0.001	R = 0.44, P = 0.019
AQP4-neuropil (AU)	R = − 0.22 (ns)	R = 0.43, P = 0.024
Dp71-perivascular (AU)	R = − 0.65, P < 0.001	R = 0.64, P < 0.001
Dp71-neuropil (AU)	R = − 0.09 (ns)	R = 0.24 (ns)
CD68 neuropil (% area)	R = − 0.36, P = 0.056	R = 0.19 (ns)

R = Pearson correlations. It should be noted that reduced arbitrary units (AU) is indicative of increased AQP4 expression because higher AQP4 causes less transmission of light and thereby reduction of AUs. On the other hand, increased AU means that more light is transferred because of reduced AQP4 expression. Ns = non-significant

**Fig. 8:**
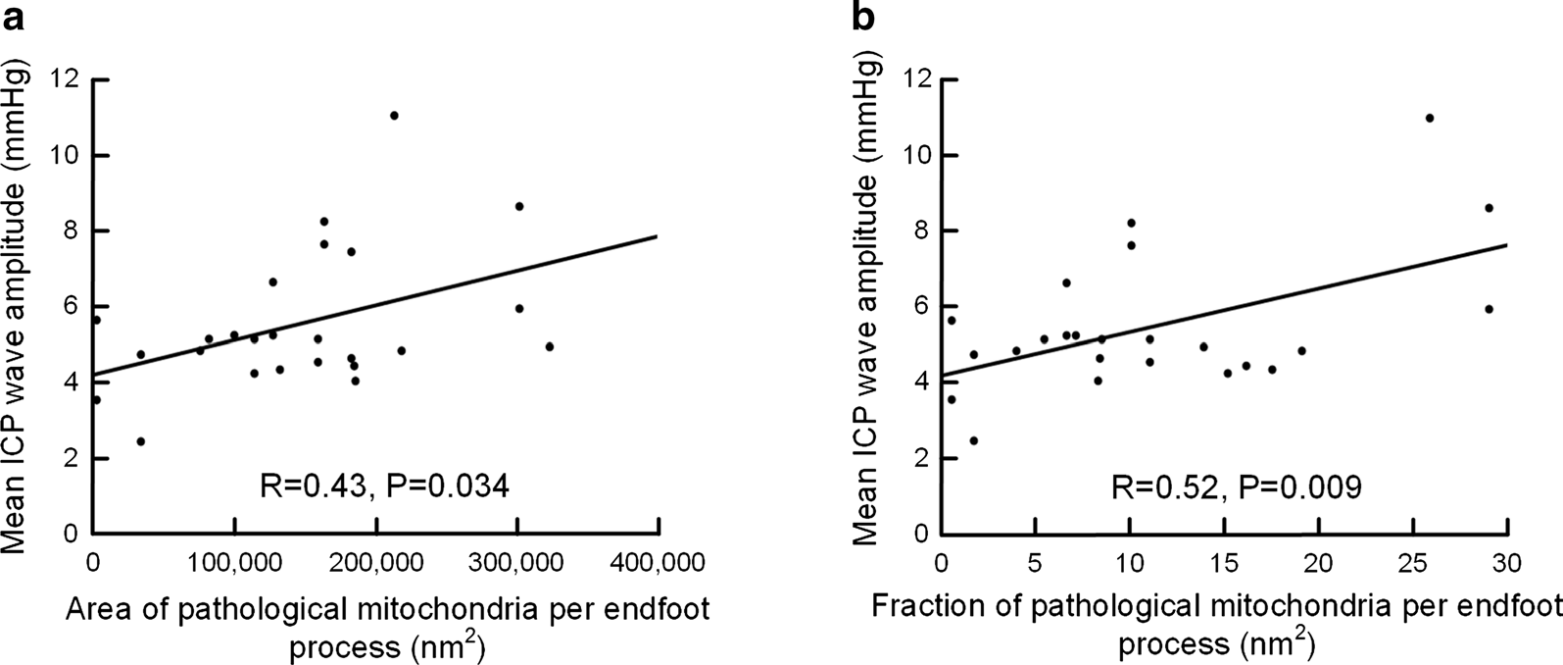
Association between pulsatile ICP and measures of pathological mitochondria in astrocytic endfoot processes in iNPH patients. **a** There was a significant positive correlation between mean ICP wave amplitude (MWA_ICP_) and area of pathological mitochondria in astrocytic endfoot processes (R = 0.43, P = 0.034). **b** There also was a significant positive correlation between MWA_ICP_ and fraction of pathological mitochondria in astrocytic endfoot processes (R = 0.52, P = 0.009). In comparison, no significant correlation was found between the static ICP (mean ICP) and either of the area of pathological mitochondria in astrocytic endfoot processes (R = 0.35, P = 0.086) or the fraction of pathological mitochondria in astrocytic endfoot processes (R = 0.37, P = 0.08)

With increasing area (Fig. 8a) and fraction (Fig. 8b) of pathological mitochondria in endfoot processes, the mean intracranial pressure wave amplitude (MWA_ICP_) was increased, while this was not seen for mean intracranial pressure (ICP) (data not shown).

### Comorbidity versus normal and pathological mitochondria in iNPH

Among the 30 iNPH patients, 14 (48%) had arterial hypertension and 10 (35%) diabetes mellitus. Except for a higher number of pathological mitochondria in the astrocytic endfoot processes of iNPH patients with diabetes (Additional file 1: Fig. S8), there were no significant differences between iNPH patients with or without comorbidity (arterial hypertension or diabetes) for any of the other reported mitochondria attributes.

## Discussion

The main observation of this study was evidence of pathological mitochondria in neuronal soma, pre- and postsynaptic terminals and astrocytic endfoot processes of iNPH patients. There were also reduced mitochondria-endoplasmic reticulum distance in neuronal soma of iNPH. These observations in combination with the presence of non-fused and accumulated autophagic vacuoles may indicate defective cellular clearance in iNPH patients. The signs of defective cellular clearance were accompanied with shortened postsynaptic density lengths in iNPH, potentially suggesting reduced synaptic activity. Finally, the results provide evidence for an association between the investigated measures of impaired cellular clearance and degree of astrogliosis as well as perivascular expression of AQP4 and Dp71, a part of the dystrophin-associated protein complex.

### The dementia subtype iNPH

iNPH is a subtype of dementia, characterized by abnormal CSF circulation homeostasis, and clinical improvement by shunt surgery, at least to some degree and for a certain period [3]. Of the iNPH patients in the present study, 27/30 individuals received a shunt. Twenty-five of these were clinical responders, qualifying for *definite iNPH*, according to the Japanese guidelines [22]. Therefore, we consider the present observations as representative for iNPH patients in general.

iNPH shares many similarities with Alzheimer’s disease. For instance, brain biopsies from iNPH patients show extracellular deposition of amyloid-β and cellular accumulation of neuro-fibrillary tangles of HPτ [5, 23], as well as other neuropathological similarities between iNPH and Alzheimer’s disease [4]. Previous MRI studies of extra-vascular movement of a CSF tracer showed delayed clearance of the CSF tracer from CSF spaces and from glymphatic pathways of iNPH patients [14–16]. While delayed extravascular clearance of a CSF tracer might be relevant for clearance of soluble amyloid-β, the link to cellular clearance of HPτ seems less obvious.

### Pathological mitochondria in neurons of iNPH patients

Mitochondria are dynamic organelles that are key producers of adenosine triphosphate, and are instrumental for maintaining cellular metabolic homeostasis, cell survival and cell death. The mitochondrial machinery consumes oxygen to generate sufficient energy for the maintenance of normal cellular processes. To protect mitochondria from damage, cells and mitochondria have developed a wide-range of mitochondrial quality control mechanisms that clear damaged mitochondrial cargo, enabling the mitochondria to repair damage and restore normal function [24].

In this study, we systematically categorized and analyzed normal, pathological, and clustered mitochondria in soma and nerve terminals of REF and iNPH patients. In neuronal soma of iNPH patients, the fraction of normal mitochondria was reduced while the numbers of pathological and clustered mitochondria were increased. This finding may be indicative of defective cellular clearance and impaired handling of damaged mitochondria in iNPH. Normally, dysfunctional and damaged mitochondria are repaired in the soma, or they become cleared by mitophagy, a process of imperative role for mitochondrial health [25]. Damaged mitochondria can also be degraded by astrocytes through translocation from neurons [26]. This process is required to maintain a functional mitochondrial biomass in cells. We also found an increased mass of clustered mitochondria in the soma of iNPH subjects, which as well may be indicative of defective mitophagy. To our knowledge, systematic electron microscopy analyses of mitochondrial health in different neuronal compartments and astrocytic endfoot processes has previously not been done in iNPH patients.

The present observations of increased occurrence of pathological and clustered mitochondria in neuronal soma further link iNPH with other neurodegenerative diseases and with Alzheimer’s dementia. In neurodegenerative diseases such as Huntington’s disease [27, 28], Alzheimer’s disease [29] and amyotrophic lateral sclerosis [30], breakdown of mitochondrial dynamics has previously been shown to affect mitochondrial biogenesis that in turn may lead to insufficient axonal mitochondrial transport. Moreover, impaired removal of damaged and dysfunctional mitochondria has been shown to facilitate pathological features of Alzheimer’s disease [31].

In iNPH individuals, we found reduced ratios of mitochondria in presynaptic terminal versus soma as well as reduced ratios of mitochondria in postsynaptic terminals versus soma. Moreover, the number of pathological mitochondria was increased in both pre- and postsynaptic terminals. These observations may be indicative of impaired mitochondrial trafficking from the nerve terminal to soma and of disturbed synaptic activity in iNPH patients. In order to maintain compartmentalized energy demands, mitochondria follow anterograde and retrograde transport, which control the mitochondrial biogenesis in axon terminals and cell bodies [32, 33]. Since the energy demand is higher in the pre- and post-synaptic terminals of neurons, sufficient number of mitochondria in the terminals is required to maintain synaptic function [34, 35]. Normally, damaged and dysfunctional mitochondria in the distal regions of the neuron are transported retrograde to the soma where they are repaired through fission and fusion mechanisms [36, 37]. The mitochondrial movement is highly associated to this mitochondrial fusion-fission machinery [38].

Breakdown of the normal mitochondrial trafficking may lead to neurodegeneration. For example, in a model of Huntington’s disease induced pluripotent stem cells (iPSCs) derived neurons, impaired mitochondrial trafficking was associated with neuronal abnormalities [39]. Abnormal mitochondrial trafficking has also been reported in other neurodegenerative diseases, such as Parkinson’s disease [40] and Alzheimer’s disease [38], which accelerates synaptic energy deprivation and oxidative stress [41]. Mitochondrial motility is severely hampered in Alzheimer’s disease due to amyloid-β and HPτ pathology. The amyloid-β pathology reduced the number of functional mitochondria in presynaptic terminal in Alzheimer’s disease [42]. The brains of Alzheimer victims show abnormal accumulation of defective mitochondria in the nerve terminal and soma [43]. Importantly, the deviation from appropriate removal of damaged mitochondria from neurons and astrocytes contribute to failure of synaptic and neuronal functions.

### Altered mitochondria-endoplasmic reticulum contact sites in iNPH patients

Another indication of defective cellular clearance of metabolites in iNPH was the evidence of shortened distance between mitochondria and endoplasmic reticulum, resulting in increased mitochondria-endoplasmic reticulum contact sites in iNPH, as compared with REF individuals. Comparable to the present findings, a previous study reported shortened distance between mitochondria and endoplasmic reticulum and increased number of mitochondria-endoplasmic reticulum contact sites per cell in iNPH patients who presented with amyloid plaques and cellular neuro-fibrillary tangles of hyperphosphorylated tau (HPτ) [44]. In addition, the authors reported that number of mitochondria-endoplasmic reticulum contact sites positively correlated with increasing age [44]. The presently reported differences in distance between mitochondria and endoplasmic reticulum between REF and iNPH individuals were significant after considering age differences.

The characteristics of mitochondria-endoplasmic reticulum contact sites play a role in Ca^2+^ signaling, mitochondrial dynamics, bioenergetics and turnover [45, 46]. In Alzheimer’s disease, mitochondria-endoplasmic reticulum contact sites were associated with amyloid-β and HPτ accumulation [47]. As previously suggested [44], the elevation of mitochondria-endoplasmic reticulum contact sites may alter Ca^2+^ homeostasis [48]. Tentatively, this mechanism might deteriorate mitochondrial function and trafficking in iNPH.

As compared with another neurodegenerative disease, amyotrophic lateral sclerosis, endoplasmic reticulum pathology was associated with defective autophagy and mitochondrial abnormalities [49].

### Accumulation of autophagic vacuoles in iNPH patients

Autophagic vacuoles were more abundant in iNPH than REF individuals. An increased amount of non-fused autophagic vacuoles may be related to defective cellular clearance of debris [50], and inefficient autophagic machinery to clean up the damaged mitochondria in the neuronal soma [31] may be one mechanism behind the elevated levels of pathological mitochondria in iNPH.

Furthermore, it may be speculated that a dumping of autophagic vacuoles in iNPH may contribute to accumulation of amyloid-β and HPτ protein in iNPH [23], as has previously been shown in Alzheimer’s disease [5, 43]. The extensive accumulation of immature autophagic vacuoles in neurites is evident in Alzheimer’s disease [51]. Since damaged mitochondrial accumulation is an early event in Alzheimer’s disease and therefore elevated levels of autophagic degradation of defective mitochondria may halt the progress of Alzheimer’s disease pathogenesis.

### Altered synaptic structure in iNPH evident by postsynaptic density

The average length of the postsynaptic densities was significantly reduced in iNPH, as compared with REF subjects. The postsynaptic density length provides a measure of the strength of the synaptic activity. It has been shown that oligomeric amyloid-β in close proximity to the postsynaptic area may shrink the postsynaptic density length, reduce synaptic plasticity and increase synaptic loss [52]. The presence of amyloid-β in iNPH patients and reduced postsynaptic density length may therefore be one contributing factor to the cognitive decline and synaptic loss in iNPH. In comparison, synaptic loss is considered to be an early event of Alzheimer’s disease [53].

Mitochondrial trafficking and distribution is connected to synaptic activity [54]. In the present iNPH cohort, we found that the number of pathological mitochondria was significantly increased in both pre- and post-synaptic terminals, and the area of normal mitochondria per postsynaptic terminal was significantly reduced. The increased mass of pathological mitochondria in the synaptic terminals may be related to insufficient energy supply, which may cause shrinkage of synaptic length that in turn leads to poor synaptic transmission and synaptic plasticity. In comparison, synaptic mitochondrial pathology is one early pathological feature of Alzheimer’s disease, which contribute to degeneration of synaptic functions [55].

### Mitochondria phenotype in the astrocytic endfeet of iNPH individuals

The present results showed altered mitochondria phenotype in astrocytic endfoot processes of iNPH individuals. The number of normal mitochondria was reduced and the number and area of pathological mitochondria increased in the endfoot processes of iNPH patients. While this is a morphological study, the data may be indicative of altered integrity of the neuro-vascular unit and blood–brain barrier function in iNPH.

Failure of the neurovascular unit may alter blood flow and result in a low-grade ischemia, which has been demonstrated previously in iNPH [56]. Astrocytes seems critical for bridging the connection between neurons and blood vessels, playing key roles in brain metabolism [57]. The astrocytic end-feet wrap up the microvasculature contributing to maintenance of blood–brain barrier function [58]. In astrocytes, the positioning of mitochondria seems to influence Ca^2+^ signaling [59]. Notably, mitochondria play a crucial role in glutamate-glutamine shuttle in astrocytes [60]. However, the spatial function of mitochondria is yet to be investigated in astrocytic processes such as in perivascular astrocytic endfoot.

Proper mitochondrial function is a key factor for astrocytic energy metabolism, and mitochondrial dysfunction may lead to reactive cortical astrogliosis [61]. A recent animal study reported that mitochondrial dysfunction halts generation of new astrocytes and increases nerve cell death [61]. Transmission electron microscopic observation showed that degeneration of astrocytic processes is associated with increased presence of swollen and abnormally shaped mitochondria seen in ischemic neuronal injury during 5 to 48 h post-ischemia [62]. Notably, the electron microscopic 3D reconstruction revealed the presence of bundle of mitochondria in the endfoot processes close to perivascular endfoot membrane [12].

In this study, we also found an association between increase of pathological mitochondria in endfoot processes and loss of perivascular AQP4 in iNPH. Previously, we reported loss of perivascular AQP4 in iNPH [17, 63]. The present observations of reduced number of normal and increased number of pathological mitochondria in astrocytic endfoot processes may be indicative of defective energy metabolism in endfoot processes of iNPH, which in turn may be associated with astrocytic malfunctions and impaired water and solute exchange. In comparison, previous ultrastructural observations in chronic compressive spinal cord injury showed swollen and disrupted mitochondria in astrocytes of spinal cord [64]. Moreover, observations in animals suggest that aging aggravates mitochondrial alterations in the astrocytic endfeet [65].

## Limitations

Our results of comparing iNPH with REF subjects could be affected by other factors than disease per se. The REF individuals were significantly younger than the iNPH patients. Moreover, the REF subjects were not healthy as 6/9 REF subjects had suffered epilepsy for several years. The occurrence of co-morbidity also differed between groups.

Even though our control individuals cannot be considered as perfect in all respects, brain tissue from normal and age-matched controls cannot be obtained for ethical reasons. Reference brain tissue is only possible to obtain from individuals undergoing brain surgery for various reasons in which removal of apparently normal tissue is required to treat the disease that is the clinical indication for neurosurgery.

We cannot exclude that age-difference between groups affected the present results to some degree. There is evidence of impaired mitochondria function with increasing age [66]. However, statistical comparisons between iNPH/REF groups were done using multivariate analysis, taking into consideration age. Based on these results, the present group-differences cannot be attributed to age alone.

From previous studies, we know that the prevalence of both arterial hypertension and diabetes is increased in iNPH [67]. In the present iNPH cohort, except for a significantly higher number of pathological mitochondria in astrocytic endfeet of iNPH patients with dementia, we found no differences in mitochondria phenotype markers between patients with or without arterial hypertension or those with or without diabetes mellitus. We could therefore not explain the present results by different profiles in co-morbidity.

## Conclusions

The present study provides several lines of evidence of altered mitochondria phenotype in neurons and perivascular astrocytic endfeet of iNPH patients. We suggest that these observations are related to defective cellular clearance in iNPH. Cellular dysregulation at the glia-vascular interface may be involved in both the cellular and extracellular clearance failure characterizing these patients. The present observations are in line with our previous suggestions that dysfunctional neurovascular units contribute to the neurodegeneration, paravascular clearance failure and CSF circulation disturbance that is typical for iNPH. The cause of clearance failure is not known. Whether or not the altered mitochondria phenotype is a primary or secondary phenomenon remains to be further elucidated.

## Supplementary information

**Additional file 1.** Additional table and figures.

## Data Availability

The data presented in this work is available upon request.

## Abbreviations

AQP4: aquaporin-4
AV: autophagic vacuole
CD68: cluster of differentiation 68
CM: clustered mitochondria
CSF: cerebrospinal fluid
Dp71: dystrophin 71
EF: endfoot process
ER: endoplasmic reticulum
GFAP: glial fibrillary acidic protein
HPτ: hyperphosphorylated tau
ICP: intracranial pressure
iNPH: idiopathic normal pressure hydrocephalus
iPSCs: induced pluripotent stem cells
MERCs: mitochondria-endoplasmic reticulum contact sites
MRI: magnetic resonance imaging
NM: normal mitochondria
PGC1α: peroxisome proliferator-activated receptor γ-coactivator-1α
PM: pathological mitochondria
PSD: postsynaptic density
REF: reference
