# Supplementary material for: Pathological mitochondria in neurons and perivascular astrocytic endfeet of idiopathic normal pressure hydrocephalus patients
Source: Fluids Barriers CNS. 2019 Dec 18;16:39. doi: 10.1186/s12987-019-0160-7 (PMC6918585; doi:10.1186/s12987-019-0160-7)
Supplement: Supplementary file 1 — Additional file 1. Additional table and figures. [file 12987_2019_160_MOESM1_ESM.pdf]

Additional Material

Pathologic mitochondria in neurons and perivascular astrocytic endfeet of idiopathic normal pressure hydrocephalus patients

Md Mahdi Hasan-Olive, MSc[1,2], Rune Enger, MD, PhD[3,4], Hans-Arne Hansson, MD, PhD[5], Erlend A. Nagelhus, MD, PhD[3,4], Per Kristian Eide MD PhD[1,2]

[1]*Department of Neurosurgery, Oslo University Hospital - Rikshospitalet, 0027 Oslo, Norway*
[2]*Institute of Clinical Medicine, Faculty of Medicine, University of Oslo, Oslo, Norway,*
[3]*GliaLab and Letten Centre, Division of Physiology, Department of Molecular Medicine, Institute of Basic Medical Sciences, University of Oslo, 0317 Oslo, Norway*
[4]*Department of Neurology, Oslo University Hospital - Rikshospitalet, 0027 Oslo, Norway*
[5]*Institute of Biomedicine, University of Gothenburg, Göteborg, Sweden*

Correspondence:
Professor Per Kristian Eide, MD PhD
Department of Neurosurgery
Oslo University Hospital - Rikshospitalet
Pb 4950 Nydalen,
N-0424 Oslo, Norway
p.k.eide@medisin.uio.no/peide@ous-hf.no
Phone +47-23074321. Fax +47-23074310

Table S1. Information about cohorts of REF and iNPH patients

	REF	iNPH	Significance
Number	9	30	
Gender (F/M)	4/5	14/16	ns
Age mean at inclusion (yrs)	42.7 ± 18.6	70.8 ± 8.9	*P* < 0.001
Co-morbidity			
Arterial hypertension, n (%)	2 (22.2%)	14 (48.3%)	ns
Diabetes mellitus, n (%)	0	10 (34.5%)	P = 0.04
Pre-operative symptoms			
Duration of symptoms (yrs.)	10.6 ± 10.5	2.4 ± 1.6	*P* < 0.001
Total NPH score	15 (14 - 15)	10 (7 - 13)	*P* < 0.001
Gait NPH sub-score	5 (5 - 5)	3 (2 - 4)	*P* < 0.001
Incontinence NPH sub-score	5 (5 - 5)	3 (2 - 5)	*P* < 0.001
Cognitive NPH sub-score	5 (4 - 5)	3 (3 - 4)	*P* < 0.001
Management			
Shunt		25 (83.3%)	
ETV		2 (6.7%)	
Conservative (no surgery) for iNPH		3 (10%)	
Cranial surgery [epilepsy (n=6), aneurysm (n=2), tumor (n=1)]	9 (100%)		
Clinical improvement NPH score during follow-up			
Improved clinical function		25/30 (83.3%)	
No improved clinical function		2/30 (6.7%)	
Lost to follow-up		3/30 (10%)	

Categorical data presented as numbers; continuous data presented as mean ± standard deviation. Significant differences between continuous variables were determined by independent samples t-test, and differences between categorical data were determined by Pearson chi square test (ns: non-significant). Differences between groups for NPH score was determined by Mann-Whitney U-test.

Fig. S1

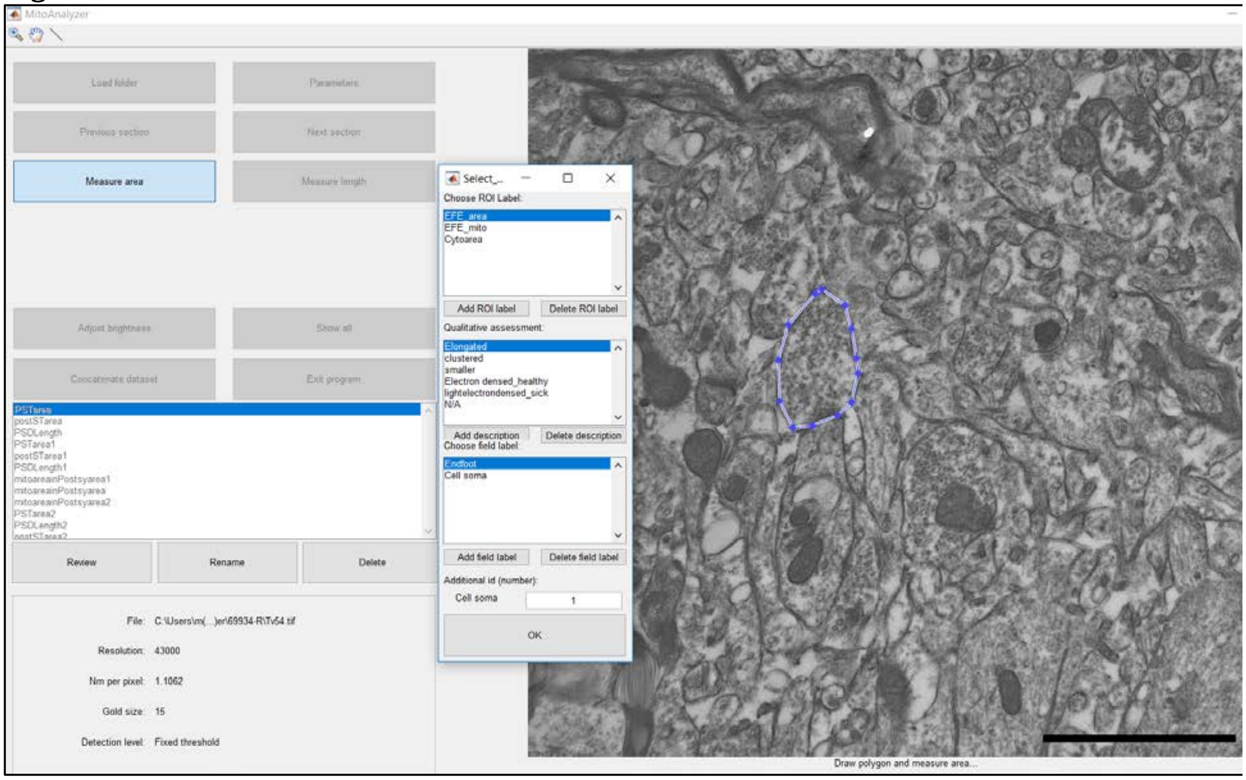

The Matlab based toolbox "Mitoanalyzer" was used to analyze different variables from neuronal soma, pre-and post-synaptic nerve terminals and astrocytic endfoot processes. The toolbox has extended features for selecting defined regions of interest (ROIs) together with qualitative assessments.

Fig. S2

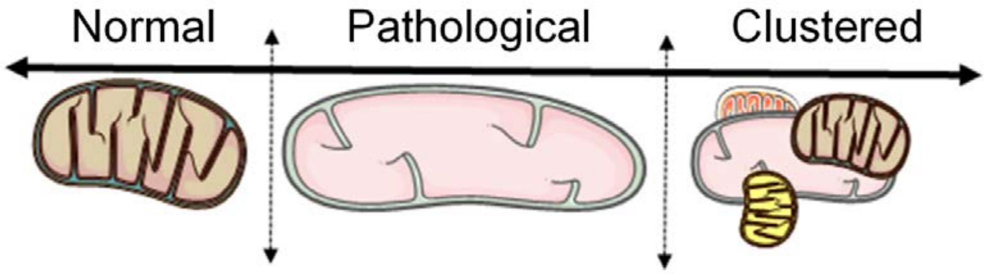

Cartoon of normal, pathological and clustered mitochondria. The normal mitochondria were dark and electron dense, with intact matrix and cristae, and regular shape. Pathological mitochondria were light and less electron dense, with less intact matrix and cristae, and irregular shape and a swollen appearance. Clustered mitochondria were clumped and aggregated due to accumulation of damaged mitochondria. Mitochondria were irregularly electron dense, with less intact cristae, and irregular shaped.

Fig. S3

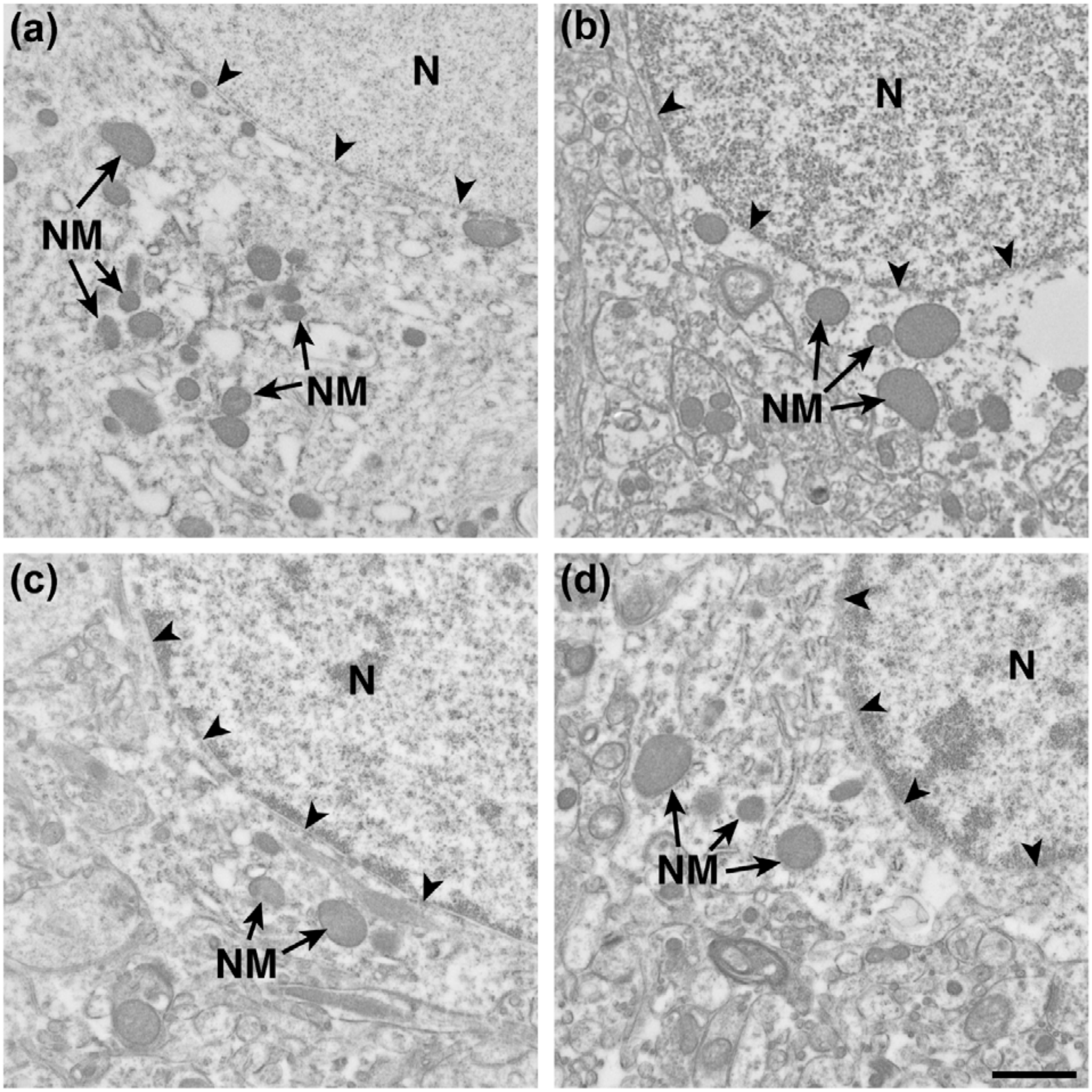

Electron micrographs showing the electron dense, regular-shaped normal mitochondria present in the neuronal soma of reference subjects. NM = normal mitochondria. N= Nucleus. Nuclear membrane: Black arrow heads. Magnification 11500 x; Scale bar 1 µm.

Fig. S4

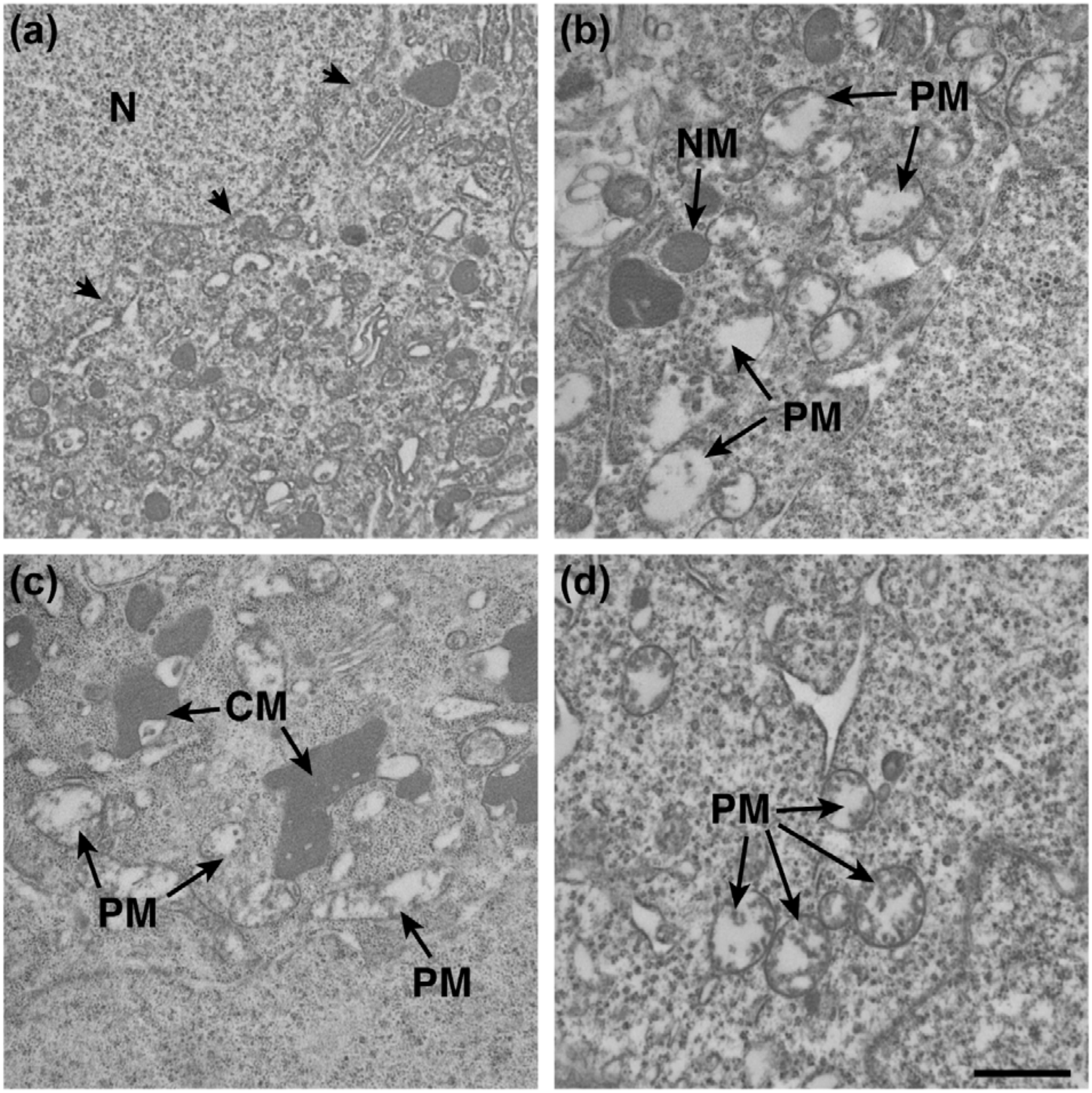

Electron micrographs showing different features of pathological mitochondria (PM) in iNPH patients. Pathological mitochondria were swollen, less electron-dense, and with less prominent mitochondrial cristae, both with low resolution (a) and higher resolutions (b-d). Normal mitochondria (NM) were electron-dense, and pathological mitochondria (PM) were less electron-dense mitochondria. Clustered mitochondria (CM) were represented by accumulation of large number of swollen mitochondria in the neuronal soma. Magnifications: (a) 6000 x; (b) 8200 x; (c-d) 11500 x; Scale bar 1 µm.

Fig. S5

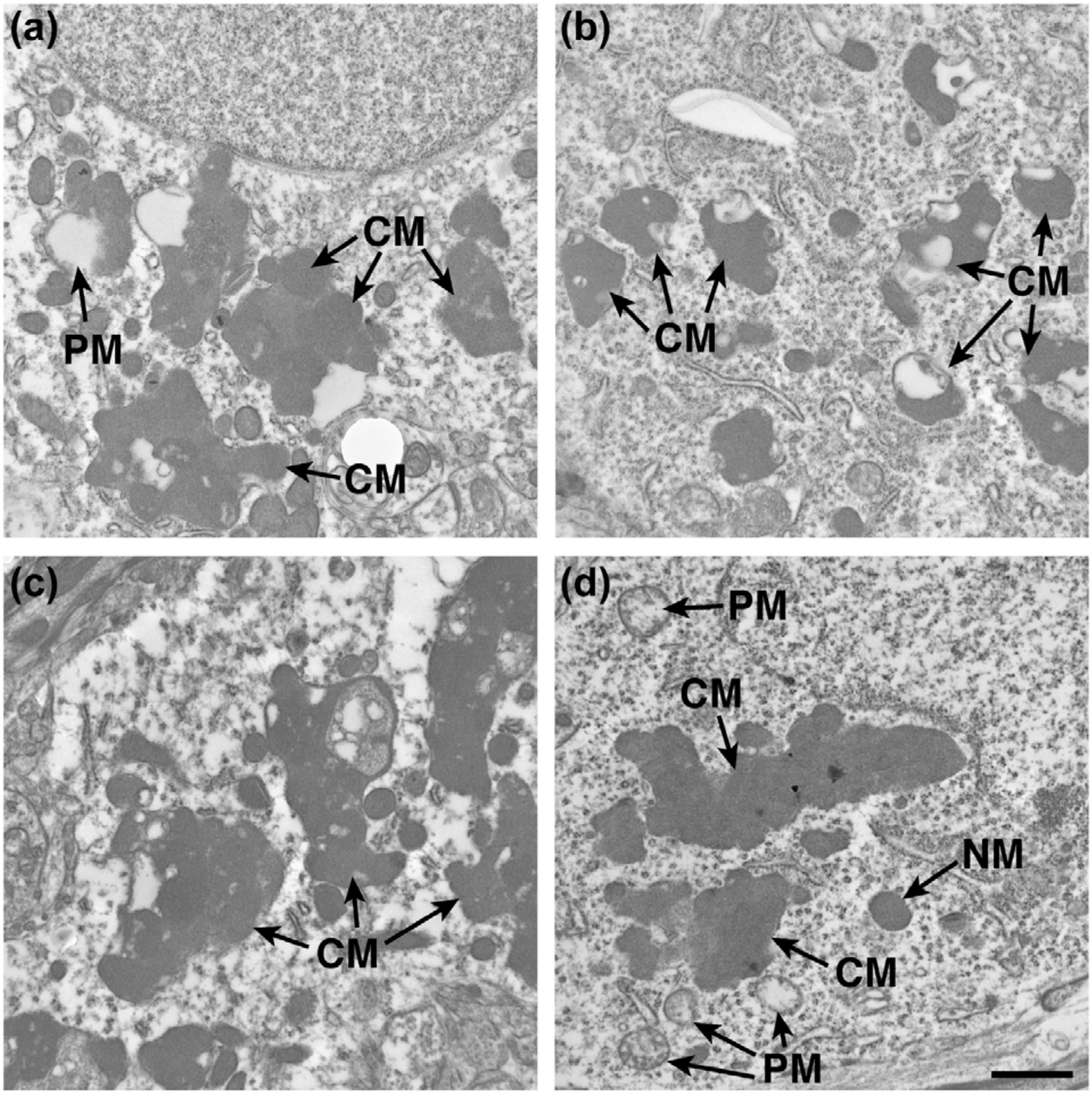

Electron micrographs showing examples of clustered mitochondria (CM) in iNPH, typically characterized by abnormal shaped and aggregated mitochondria in the neuronal soma. Note the co-presence of clustered and pathological mitochondria (PM). Magnification: 11500 x; Scale bar 1 µm.

Fig. S6

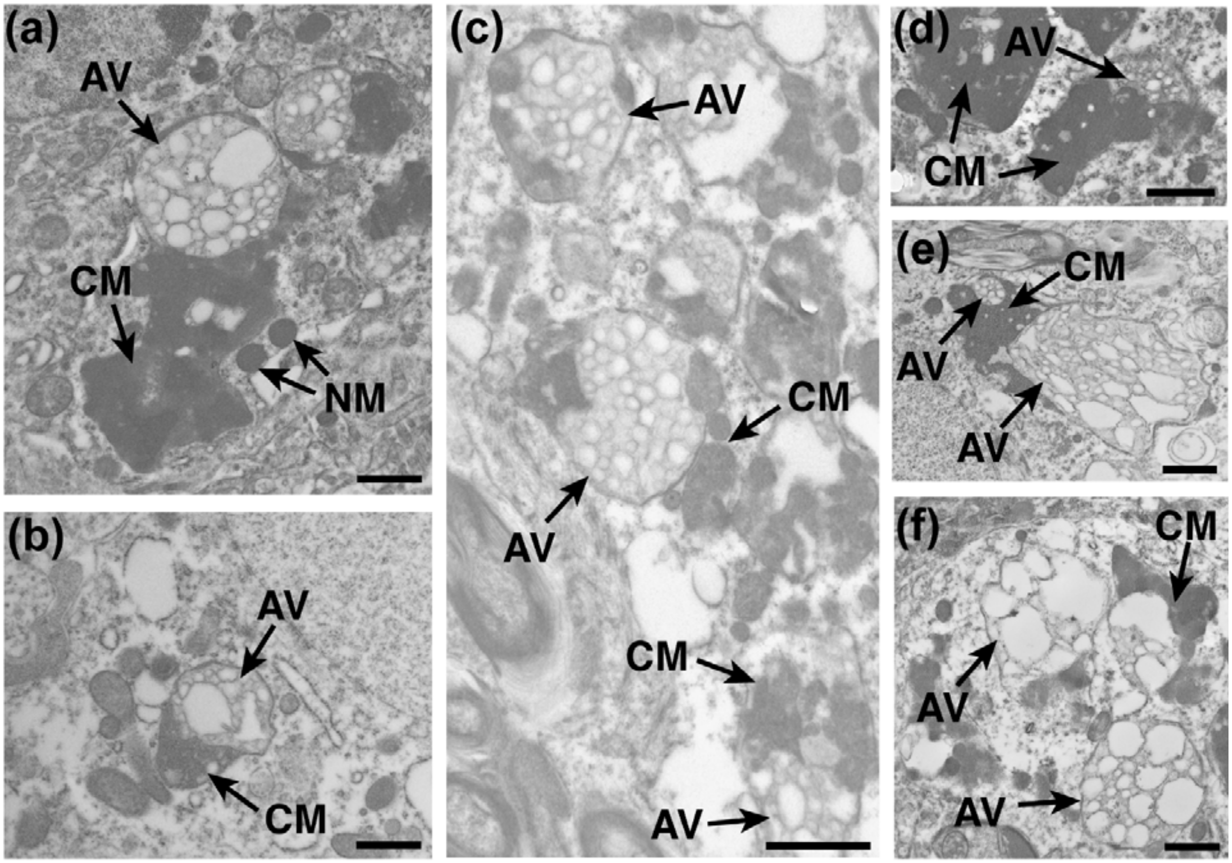

Electron micrographs showing the presence of autophagic vacuoles (AVs) in iNPH patients. The AVs are in very close proximity with clustered mitochondria (CM). The non-fused AVs in the soma represent defective signs of intracellular clearance in iNPH. NM: Normal mitochondria. Magnification 11500 x; Scale bar 1 µm.

Fig. S7

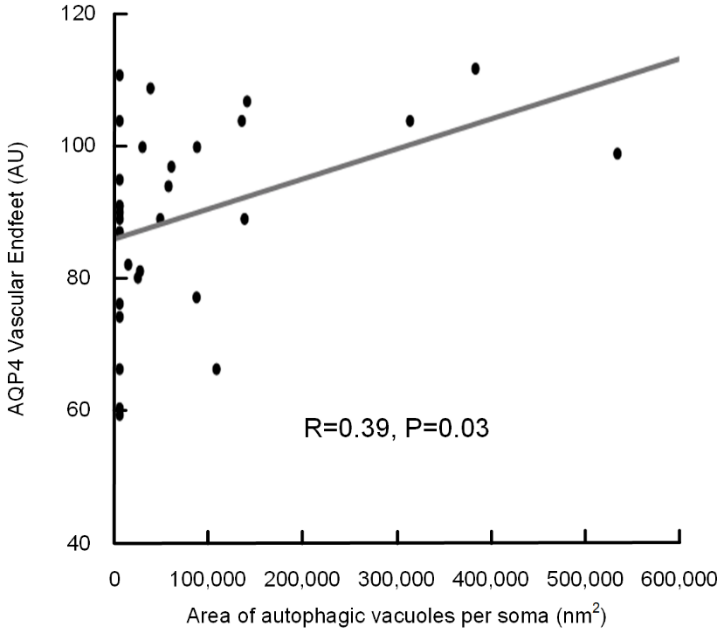

Significant correlation between area of autophagy vacuoles in soma and perivascular expression of AQP4. Increasing arbitrary units (AU) of AQP4 at vascular endfeet means reduced perivascular expression of AQP4. Therefore, reduced perivascular expression of AQP4 was associated with increased area of autophagy vacuoles.

Fig. S8

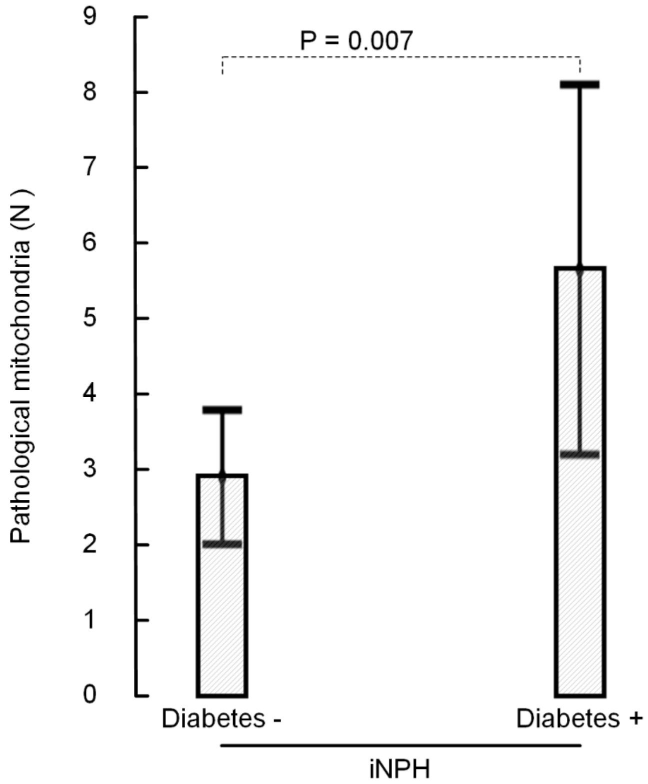

Significantly increased number (N) of pathological mitochondria in astrocytic endfeet of iNPH patients with diabetes mellitus (independent samples t-test). Error bars are 95% CI.
